# Blood pressure monitoring techniques in the natural state of multi-scenes: A review

**DOI:** 10.3389/fmed.2022.851172

**Published:** 2022-08-26

**Authors:** Ziyi Liu, Congcong Zhou, Hongwei Wang, Yong He

**Affiliations:** ^1^College of Biosystems Engineering and Food Science, Zhejiang University, Hangzhou, China; ^2^Guangdong Transtek Medical Electronics Co., Ltd., Zhongshan, China; ^3^Sir Run Run Shaw Hospital, School of Medicine, Zhejiang University, Hangzhou, China; ^4^Biosensor National Special Laboratory, College of Biomedical Engineering and Instrument Science, Zhejiang University, Hangzhou, China; ^5^Tongde Hospital of Zhejiang Province, Hangzhou, China

**Keywords:** wearable, blood pressure, multi-scenario, natural state, finger

## Abstract

Blood pressure is one of the basic physiological parameters of human physiology. Frequent and repeated measurement of blood pressure along with recording of environmental or other physiological parameters when measuring blood pressure may reveal important cardiovascular risk factors that can predict occurrence of cardiovascular events. Currently, wearable non-invasive blood pressure measurement technology has attracted much research attention. Several different technical routes have been proposed to solve the challenge between portability or continuity of measurement methods and medical level accuracy of measurement results. The accuracy of blood pressure measurement technology based on auscultation and oscillography has been clinically verified, while majority of other technical routes are being explored at laboratory or multi-center clinical demonstration stage. Normally, Blood pressure measurement based on oscillographic method outside the hospital can only be measured at intervals. There is a need to develop techniques for frequent and high-precision blood pressure measurement under natural conditions outside the hospital. In this paper, we discussed the current status of blood pressure measurement technology and development trends of blood pressure measurement technology in different scenarios. We focuses on the key technical challenges and the latest advances in the study of miniaturization devices based on oscillographic method at wrist and PTT related method at finger positions as well as technology processes. This study is of great significance to the application of high frequency blood pressure measurement technology.

## Introduction

Blood pressure (BP) is one of the basic physiological parameters of human physiology. Currently, in the main international guidelines, ambulatory blood pressure monitoring (ABPM), and/or home blood pressure monitoring (HBPM) are recommended for the diagnosis of hypertension. They are mainly carried out using electronic sphygmomanometer based on oscillometry. ABPM can complement HBPM when there is uncertainty around threshold values or there is disagreement. Studies have shown that daily BP monitoring can help to predict mortality and routine management of BP of most known or recessive hypertensive patients (1–3). Continuous BP monitoring can reduce the incidence and mortality of cardiovascular diseases. In recent years, wearable non-invasive blood pressure measurement (BPM) technology has been investigated as an important home-based daily BP monitoring technology. Numerous technical routes have been established to solve the challenge of portability or continuity of measurement methods and the accuracy of measurement results at the medical level. Studies are needed to determine how the frequent and high-precision BPM under natural conditions outside the hospital can be achieved (4–7).

## Scene based blood pressure monitoring techniques

**FIGURE 1:**
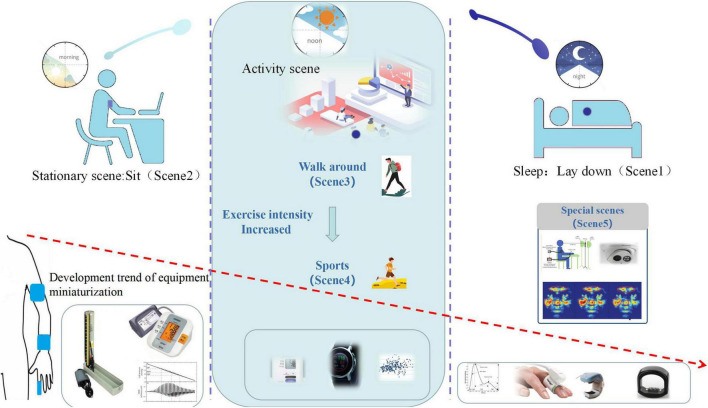
Schematic diagram of scene based blood pressure monitoring. Timeline of 1 day from left to right describes morning to night. The descending arrow describes the trends of miniaturization devises and monitor positions from up arm to finger. The two vertical dotted lines distinguish stationary scene, activity scene and sleep scene, the typical monitoring devices are shown accordingly.

Regarding limitation of the measurement accuracy of the current biomedical sensing measurement technology of human movement, this paper divides the scenes of the human body from rest to intense movement into four levels as displayed in Figure 1. Various personalized BPM technologies including non-contact, wearable, and portable have been studied for the above five states. In scene 1 state, the human body is mainly lying flat and maintain still. At this time, the heart and the human arm are basically on the same horizontal plane, which attains ABPM or timing BPM. In scene 2 state, the human body is in a sitting position, i.e., a typical HBPM scene, auscultation or cuff sphygmomanometer is commonly used to measure BP. In scene 3 state, the human body is in a state of walking which does not meet the basic condition demands for BPM. Normally, researchers detect BP in the resting state during walking. Under the scene 4 condition, the human body carries on physical training, with vigorous movement and active metabolism, only parameters including heart rate can be monitored and the BP is difficult to detect. Scene_ spec state is a special state, where new measurement technology solutions including visual, imaging module are applied.

When an individual is sleeping at night (Scene 1), BPM technique based on the principle of volume clamp method and pulse wave velocity (PWV) method achieves continuous BP monitoring while the sleep quality is high. A meta-analysis by Saugel demonstrated that, in addition to surgical or critical patients, the use of non-invasive fingertip cuff-based constant volume methods to measure arterial BP and cardiac output parameters is interchangeable with invasive standard methods (8). It normally requires the test object to remain quiet, and single BPM is performed by auscultation or oscillometry (9). Luo et al. (10) integrated a flexible piezoresistive sensor (FPS) and body surface electrocardiograph (ECG) sensor to design a wearable sensor placement system, which restored signal monitoring within a few seconds after local interference stops, and used the pulse transit time (PTT) method to achieve step-by-step BPM.

While we mainly consider the situation that people need sedentary or ambush work (scene 2). The duration of sedentary is related to the decrease of cardiac autonomic regulation function (11–14). Sedentary for 6–8 h potentially induces hypertension and impaired arterial function of lower limbs. A study by Silva et al. (15) found that breaking the sitting position with isometric exercise did not improve or prevent cardiovascular impairment. Unlike scene 1 state, this scenario faces more local movement interference including typing, scratching, etc., which increases the difficulty of continuous BP monitoring.

When an individual is walking (scene 3) in a mall/park, statistical methods can be used to analyze the trend of BP changes in the quiet state of the activity gap (16). Ningsih et al. showed that walking reduced systolic blood pressure (SBP) and diastolic blood pressure (DBP) in patients with hypertension, and it was used as an independent nursing intervention. Zhang et al. (17) analyzed the relationship between exercise or exercise-related behavior and BP control, the outcomes showed that walking and 60–120 min/day exercise time were the primary factors for BP control in hypertensive patients. Li et al. (18) monitored BP before walking as the standard value to study the wearable skin photoelectric system with motion artifact suppression function for sleeveless continuous BP monitoring, and the BP trend during walking was evaluated based on volume pulse wave technology.

When an individual is in an exercise/fitness scenario (scene 4), exercise-induced cardiac output increases compared to scene 3, this is associated with increased SBP and DBP (19). The significance of monitoring BP in this scenario is that regardless of the resting BP, excessive sub-maximal exercise BP causes adverse cardiovascular events or even sudden death, and exercise BP responses help identify high-risk individuals (20). Due to the increased exercise intensity, currently, measurable parameters mainly including heart rate and oxygen saturation. BP was measured before, during, and after exercise *via* auscultation or oscillometry (21, 22). Non-contact BPM based on image-based methods is required when the individual need to carry out BPM in certain special situations (Scene_spec) including skin damage or inability to wear a wearable device (5, 23). Rong and Li (24) proposed a non-contact sphygmomanometer system based on image photoplethysmography (PPG), the results showed that the system can potentially replace the conventional cuff sphygmomanometer. Golberg et al. (25) developed a remote optical system that depended on the time analysis of the secondary reflection speckle pattern to calculate the PTT of the object and estimated the BP.

Researchers have studied and developed various BPM technologies suitable for different scenarios as Table 1 demonstrates. Horizontal comparison of the technologies with each other including measuring position, cuff, cost, instrument accuracy, technology maturity degree, advantages, and disadvantages are presented. The traditional auscultation method is considered as the “gold standard” for the comparative study of oscillographic method and PWV/PTT/PAT based BPM method. The measurement results may influenced by the personal experience of experts, resulting in certain deviations. Since this method requires clinical expert knowledge, it is unsuitable for self-detection at home. Unlike other BPM technologies, the oscillometry and BPM methods based on PWV/PTT or pulse arrival time (PAT), have an extensive application prospect (26). Since the arm-cuff oscillographic method requires the use of a large cuff for inflation and deflation, the measurement of BP during sleep easily influences the sleep quality of users, leading to less comfort. At present, one of the vital directions of its development is designing a wearable device that integrates small airbags, miniaturized air pumps, valves, and other components in the wrist to improve the comfort of measurement on the premise of ensuring the measurement accuracy. The wrist-cuff systems has less influence on sleep quality and may be more comfortable than systems associated with arm-cuff system (27).

**TABLE 1:** A comparative point of view for different blood pressure (BP) monitoring methods in multi-scenes.

Applicable scene: scene 1 (A): lay down, scene 2 (B): sit, scene 3 (C): walk around, scene 4 (D): sports, scene 5 (E): special scene; Monitoring method: I: continuous monitoring, II: single measurement, III: interval measurement, take measurements every once in a while;									
**Method**	**Applicable scene**	**Monitoring method**	**Measuring position**	**Cuff**	**Cost**	**Instrument accuracy**	**Technology maturity degree**	**Advantages**	**Disadvantages**
Auscultatory (33–35)	B, C, D	II, III	Bicep	Yes	Low	Gold standard for standard control	Mature	Commonly and widely used by the medical doctors	1. User dependent, supervision form professional is necessary. 2. More than 3 min time delay between successive measurements.
Oscillometry	A, B, C, D	II, III	Bicep, Wrist	Yes	Medium	Medical Level	Arm-cuff oscillographic method is mature; Wrist-cuff systems needs more clinical validation	Most widely used method in many scenes, results are considered to be medical level	1. Uncomfortable, noisy for long time monitoring. 2. Cuff dependent.
Applanation tonometry (36–43)	A	I	Wrist	No	High	Medical Level	Mature, sensor structures are improving	Used for continuous blood pressure monitoring in Scene 1 only under supervision	1. Strict conditions for sensor location or structure and monitoring environment. 2. Supervision form professional is necessary.
Volume clamp method (44)	A	I	Wrist, Finger	Yes	High	Medical Level	Mature, control algorithms are improving	Instantaneous, continuous measurement in Scene 1 in hospitals	Complex and precise control systems are required.
PWV/PTT/PAT	A, B, C, D, E	I, II, III	Proximal and distal pulse waveform	No	Low	Accuracy is controversial	Improving, one of the hot spots of current researches	Have the potential to be used in all the scenes mentioned in this article	Calibration is required, more clinical data is needed.

Specifically, from the perspective of the universality of applicable scenes in natural conditions, the applanation tonometry and volume clamp method are generally used for continuous BP monitoring in hospitals or beat- to-beat BP monitoring. These methods usually require professional medical staff to operate. To improve the user experience based on volume clamp method, the control algorithms are improving. Chen and Chen (28) proposed a neural network predictive controller to replace the traditional Proportional-integral-derivative (PID) controller. Fortin et al. (29) proposed an innovative continuous non-invasive hemodynamic monitoring technology (CNAP2GO). Blood pressure is directly measured by the volume control method, which is integrated into the finger ring to achieve accurate continuous monitoring. Due to the complexity of the device and the dependence of accuracy on the test process, it is presently difficult to use in communities or families. PWV/PTT/PAT (Since the principles are similar, PTT is used as a representative to replace this type of methods in the following) based BPM have a wide application prospect in the measurement of BP in various scenarios. Besides, it has the potential to perform convenient measurement and continuous beat-to-beat BPM. At present, studies have performed clinical trials, while compared with oscillometry, additional clinical studies are necessary.

In conclusion, the technology maturity degree of auscultatory, applanation tonometry, volume clamp method are mature, and new control methods of volume clamp method are developing. As to oscillometry, the latest wrist-cuff systems are attractive and more clinical validations are performing. PTT related method is one of the hot spots of current researches, it is low cost and has the potential to be applied in all the scenes mentioned in this paper after calibration to achieve medical level instrument accuracy. This paper focuses on the key technical challenges and the latest advances in the study of miniaturization devices based on oscillographic method at wrist and PTT related method at finger positions as well as technology processes (30–32).

## Research and advance in wrist/finger blood pressure measurement

### Technologies based on oscillometry

The accuracy of HBPM using wrist devices has not been conclusively determined (44). In the study from Komori et al. (45), which involved 50 volunteers with normal BP and hypertension, they showed that BPM using wrist monitors provided comparable findings to those from arm monitors in non-bed conditions. Kuwabara et al. (46) reported that when Omron HEM-9601T was used in sitting postures with wrists at the heart level, measurement results met the validation criteria of the ANSI/AAMI/ISO 81060-2: 2013 Guide, with an acceptable accuracy in supine positions and roughly equivalent to sitting postures. Wrist-mounted BPM devices may lead to higher BP values, which are associated with measurement methods than arm with BP values (47–49) or deviations from standard measurement devices (50). Nevertheless, placing the airbag on the wrist is still a better option (51). Analysis of the technical scheme of wrist oscillometry BPM revealed that the main challenges include good airbag structure design, standard equipment production and matching algorithm model research (52, 53).

#### Air bag structure design for blood pressure measurement

The American Heart Association guidelines suggest that BP cuffs should be 80% of the circumference of the user’s arm and 40% of the width (54). Due to conical upper arms, obese people exhibit inaccurate BPMs when conventional cuffs are used (55–58). When cuff width decreases, BPM accuracy reduces because it may not be possible to ensure complete occlusion of wrist radial and ulnar arteries during cuff inflation (48, 59). Therefore, it is important to establish a good airbag structure design for current measurement site in assessment of miniaturized BPM devices.

**FIGURE 2:**
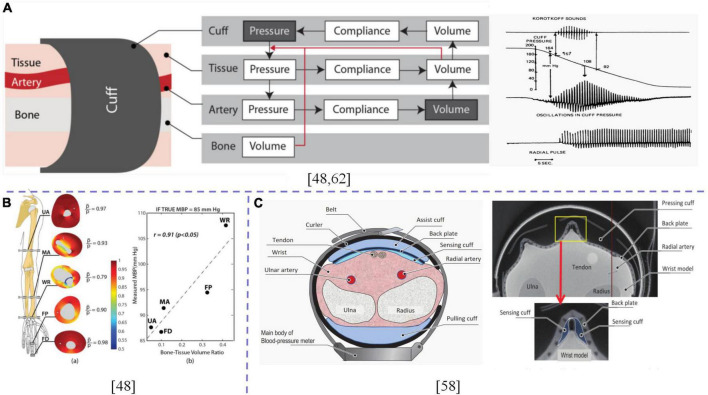
**(A)** Working principle and chain of events of cuff-based oscillometric BPM; **(B)** Simulation of pressure distribution in the tissue, scatter plot of the measured MBP and bone-tissue volume ratio when the true MBP was assumed to be 85 mmHg; **(C)** Cross-sectional view of cuff structure. X-ray CT image of a sensing cuff bent under pressure of approximately 300 mmHg when a wrist simulator was compressed. The dotted lines are used to distinguish between different contents.

Generally, studies on structural sizes of the airbag are based on hydrodynamic pressures (60), assuming that the arm can be approximated as a rotating symmetric long cylinder of linear elastic and incompressible materials, including rigid bones and tissues. It is necessary to clarify under what circumstances the arterial pressure is equal to static pressure applied at the balloon. Ursino and Cristalli (61) proposed a biomechanical model for simulating pressure transfer from the air sac to the brachial artery in the soft tissue of the arm, and evaluated the accuracy of BPM under positive/negative wall pressure. Liu et al. (49) established a bone tissue model by assuming that the limb segment wrapped in cuffs was a thick cylinder of a rigid body mixed with tissues. They simulated pressure distribution in the tissue (fat was simplified as part of a homogeneous arm tissue) and evaluated the feasibility of BPM using a small balloon structure. The traditional cuff design uses a single cuff to surround the test site. Recently, OmRon (59) designed a technical route that assigns the wrist artery compression function and the wrist surface pressure detection function to two independent cuffs, focusing on solving the challenge that the narrow cuff requires better internal pressures to block the artery and reduce the robustness of pressure shock wave as displayed in Figure 2.

An important basis for implementation of oscillographic BPM techniques is that the artery is at zero transmural pressure, and blood volume pulsation is at its maximum. At this time, it is assumed that extravascular pressure is equal to cuff pressure, while the cuff pressure corresponding to the maximum cuff pressure oscillation is equal to mean pressure (62, 63). A good balloon design is aimed at realizing zero-span wall pressure. Whether single or multiple balloons, it is necessary to consider the physiological structure of the position applied by the balloon. Under good comfort, arterial closure of the position to be tested is effectively realized. On this basis, the size design of the balloon has the potential for miniaturization (9, 27, 64).

#### Production of standard devices/simulators and algorithmic models

**FIGURE 3:**
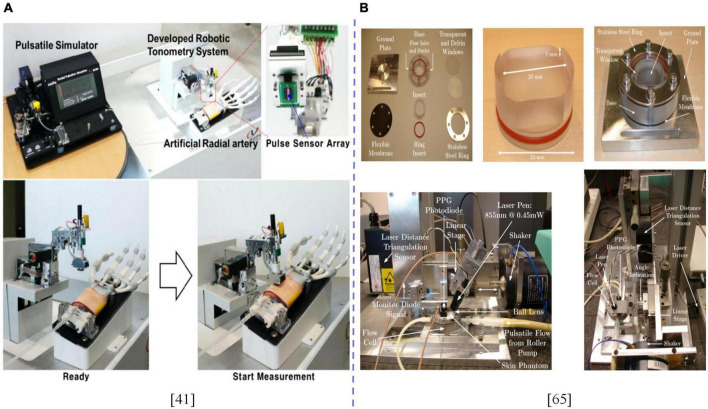
**(A)** Experimental setup of a robotic applanation tonometry pulse sensor system for acquiring pulse signals from an artificial radial artery. **(B)** Experimental setup built to measure PPG distorted by optical motion artifacts in a controllable, reproducible manner. The dotted line is used to distinguish between different contents.

When verifying the accuracy of a sphygmomanometer, the standard device/patient simulator can be used in studies on pressure calibration, repeatability and stability (50, 65). However, with advances in miniaturization design technologies, the size and structure of measured parts that can be simulated by existing simulators cannot meet the ideal requirements. Existing simulators usually simulate rigid body situations, and there is a certain gap between them and practical applications. This gap should be evaluated *via* clinical studies to improve BPM accuracy from a statistical perspective. Jun and Kim (41) developed a mechanical flat tension pulse sensor system that can easily locate and pressure the manipulator with simulated blood vessels and tissue skins to simulate wrist pressures of humans. The flat tension and oscillometric methods have contributed to advances in development of BPM technologies. Wijshoff et al. (66) designed a device to simulate human skin and blood pulses to evaluate the interference of volume pulse waves as displayed in Figure 3. Therefore, we postulate that establishment of standard devices/simulators that are in line with human physiology play an important role in development of BPM technologies, which can promote integration of BPM technologies (52), improve BPM accuracy in different scenarios, shorten clinical research time, and reduce costs associated with industrialization and technological transformation.

With advances in computing abilities of electronic systems, the significance of the algorithm model has been evaluated. Forouzanfar elucidated on the oscillographic BP estimation algorithm, including the traditional maximum amplitude and derivative oscillation methods, learning algorithm, model-based algorithm, and the algorithm based on pulse shapes and pulse passing time analysis to classify and describe various underlying algorithms (67). Tian et al. (52) fused the information of wrist pressure pulse wave and finger volume wave by using the characteristic that finger volume wave can provide BP change information. Based on the neural network model of curing parameters after training, Tian improved the accuracy of wrist BPM. Lee et al. (68) proposed a hybrid method composed of non-parametric bootstrap and machine learning technology to obtain the feature ratio used in BP estimation algorithm to improve systolic and diastolic BP estimation accuracy and to obtain the confidence interval. Alghamdi et al. (69) divided the waveforms obtained *via* the oscillographic method into three cycles and used k-nearest neighbor (kNN), weighted k-nearest neighbor (WkNN) as well as Bagged tree algorithm to classify them. A machine learning BP estimation method that is based on waveform classification was proposed, the direction of algorithm development is to model individual parameters based on the population statistical model.

### Technologies that are based on volume clamp and pulse transit time-related methods

Although hands and wrists occupy a small part of spatial volumes in the human body, they are human body carriers that enable the completion of fine movements. Relatively, the finger has an abundant capillary network, the original signal of the detected volume wave has higher signal-to-noise ratio and perfusion index, and the smaller balloon structure can be used to detect pressure waves. Detection of pulse wave signals in PTT at this site has broad application prospects in BPM in various scenarios (49, 70).

#### Theoretical models and engineering implementations of volume clamp

**FIGURE 4:**
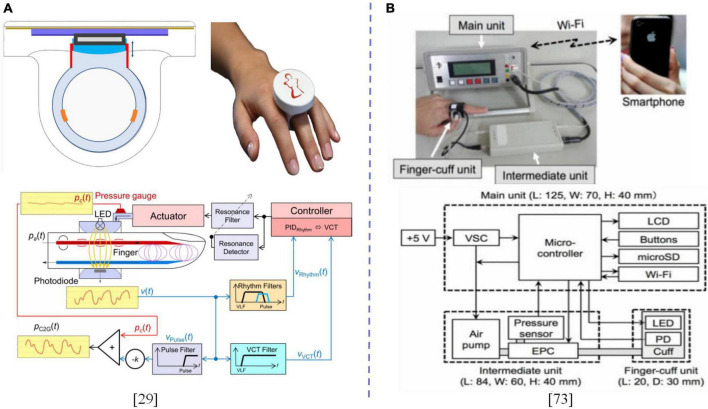
**(A)** Design of the CNAP2GO finger-ring with proposed block diagram and signal flow. **(B)** Image and block diagram of the prototype standalone instantaneous BP monitoring system. The dotted line is used to distinguish between different contents.

Blood pressure measurement based on the constant volume method and its evolution method at the fingertip is characterized with small balloon volume and high consistency with the average pressure measured by the upper arm. Various studies on the fingertip have been performed (71, 72). Gizdulich et al. (72) modeled pulse wave distortions and pressure drops between brachial and finger arteries, which was simulated by a single resonance model. A meta-analysis by Saugel et al. (8) revealed interchangeability between arterial pressure and cardiac output/cardiac index as measured by the fingertip cuff technique and invasive reference method. Schumann et al. (58) verified that consistency between finger cuff and intra-arterial measurements is better than that between oscillographic and intra-arterial measurements in obese patients. Theoretically, Liu et al. (49) established a bone tissue model to assess the feasibility of fingertip oscillometry. Panula et al. (73) evaluated the feasibility of using a linear brake consisting of a small DC motor and a gear to generate torque to compress the small artery at the fingertip. Based on the oscillographic method and peripheral-arterial BP transfer equation, BP can be measured and integrated into a finger clip pulse oximeter probe. Matsumura et al. (74) designed an improved algorithm to overcome the challenge associated with measurement errors and to improve the stability of detection in view of the error caused by spatial and organizational movements of the PPG sensor in the volume compensation method. Fortin investigated a BPM method that is suitable for integration in the finger ring. While ensuring measurement accuracy, it improves user experience and can simultaneously calculate various hemodynamic parameters, such as cardiac output, systemic peripheral resistance, and baroreceptor reflex sensitivity (29) as displayed in Figure 4.

The above frontier studies were based on various aspects, such as physiological bases, theoretical models as well as engineering realization, and discussed the feasibility of designing a miniaturized system of finger ring, finger clip or finger sleeve type based on the constant volume method or improved method at the finger end and the accuracy of BPM, which made a useful exploration for the development of wearable devices that are suitable for multiple scenes and multiple populations.

#### Development of pulse transit time-related blood pressure measurement methods

The methods associated with pulse waves and propagation speed discussed in this paper includes: i. Modeling method, which features extracts from the pulse wave morphology or the entire pulse wave as an input signal; ii. Polynomial regression modeling method, in which the pulse wave transfer time is calculated based on the peak value or other characteristic sites of the pulse wave and single-lead ECG; iii. Propagation time, which is based on pulse waves and other signals other than single-channel ECG (75). A BPM technology that is based on finger pulse wave and propagation time is a hot topic in the development of non-invasive BPM technologies. A typical technical route is to establish a multivariate regression model that is based on findings from the correlation between PTT and SBP or from morphological characteristics of the pulse wave. Another typical scheme is based on machine learning or deep learning models, using pulse wave or multidimensional features extracted from it as inputs to predict BP trends as shown in Table 2. The subject number of machine learning models are usually larger than that of traditional polynomial models.

**TABLE 2:** The machine learning models and traditional polynomial models.

	References	Parameters	Detail methods	Number of subjects	MAE (mmHg)		Standard deviation (mmHg)		Dataset	Subject description
					DBP	SBP	DBP	SBP		
Machine learning related models	Chowdhury et al. (84)	PPG features	Gaussian process regression	219	3.02	1.74	5.54	9.29	Public data (87)	657 PPG signal samples from 219 subjects.
	Aguirre et al. (86)	PPG wave	Deep learning with attention mechanism	1100	6.57	14.39	8.43	17.87	MIMIC-III	10,696 segments corresponding to 1131 subjects.
	Xing et al. (83)	PPG features, BMI	Random forest algorithm	1249	2.3 (Young, Fitting error)	2.1 (Young, Fitting error)	9.5 (Young)	13.6 (Young)	Specific data	A total of 2358 measurements were recorded, including young, old populations. Normal, pre-hypertension and stage I, II, III hypertension.
	Watanabe et al. (85)	PPG features	Specific algorithm based on second derivative of photeplethysmogram wave.	887	NA	NA	NA	NA	Specific data	A total of 887 participants were enrolled. Various feature parameters of pulse wave participants at rest an under exercise, mental stress are collected.
	Zhang et al. (30)	PTT (HRV, ECG, PPG, other PPG features)	LR: linear regression SVR: support vector regression RF: random forest regression Adaboost: adaptive boosting	3337	5.35	10.03	4.5	7.96	MIMIC I and VitalDB	Hybrid dataset (including 3,337 subjects) combining MIMIC and VitalDB databases.
Traditional polynomial models	Ghosh et al. (79)	PTT (R peak of ECG and peak of PPG)	$B\,P=\frac{a}{P\,T\,T}+b$	14	6.64 (Recumbent)	4.6 (Recumbent)	5.2 (Recumbent)	9.6 (Recumbent)	Specific data	14 subjects performed activities including: recumbent, seated, standing, walking, cycling, need calibration.
	Huynh et al. (80)	PTT	$D\,B\,P=D\,B\,P_{0}+\rho \,\frac{D^{2}}{P\,T\,T^{2}}\,1\,n\,\left[1+K\,\left(Z_{\max0}-Z_{\min}\right)\right]$(A.2a) $S\,B\,P=D\,B\,P_{0}+\rho \,\frac{D^{2}}{P\,T\,T^{2}}\,1\,n\,\left[1+K\,\left(Z_{\max0}-Z_{\min}\right)\right]$(A.2b)	15	5.02 ± 0.73 (RMSE)	8.47 ± 0.91 (RMSE)	NA	NA	Specific data	15 young, healthy human subjects leveraging handgrip exercises.
	Esmaili et al. (81)	PTT	$\text{BP}=a_{0}+\sqrt{a_{2}+a_{2}\,\frac{1}{{\mathrm{PTT}}^{2}}}$	32	3.97	6.22	5.15	9.44	Specific data	32 healthy subjects in the age range of 21–50 years performed physical exercise.
	Lin et al. (82)	PTT (PPG features)	Linear regression and four previously reported models (88–91).	22	3.16 (DS)	3.19 (DS)	5.04 (DS)	7.8 (DS)	Specific data	22 subjects when they performed mental arithmetic stress and Valsalva’s manoeuvre tasks that could induce BP fluctuations.

Chen et al. (76) and Poon and Zhang (77) elucidated on representative BPM techniques that were based on pulse wave propagation time while Ding and Zhang (78) summarized the physiological basis and formula derivation of this technology, and discussed it from the perspectives of PTT definition, calculation method, sample composition, and adopted standards. Cardiac output and peripheral arterial resistance were established to be the main factors involved in regulation of BP and arterial BP. Based on this method, Ghosh et al. (79) assessed the accuracy of BPMs under typical postures of lying, sitting, standing, walking, and cycling in different daily life scenes. They found that BP estimation based on PTT had a high accuracy in the static state while accuracy in motion state needed further improvements. Huynh et al. (80) assessed the effectiveness of PTT blood pressure measurements in the context of wrist grip training, and found that PTT-IPG-based BPM was highly accurate. Esmaili et al. (81) monitored BP changes in supervised physical exercise scenarios, collected the original signals and reference BP levels of participants, introduced a new non-linear regression model of BPM that was based on the elastic cavity theory, and achieved good results. Lin et al. (82) monitored BP fluctuations *via* two tasks of mental arithmetic stress and Valsalva’s manoeuvre test, and detected BP changes using the PTT regression model. They reported an improved measurement accuracy. These studies simulated different daily life scenarios, monitored the corresponding BP fluctuations, and predicted blood pressures based on simple linear polynomial and non-linear regression models, which verified the feasibility of the technology in laboratory environments.

Xin et al. (83), Chowdhury et al. (84), and Watanabe et al. (85) extracted various characteristic parameters from PPG signals. Based on public data sets or data sets obtained during research, the random forest algorithm, Gaussian Process regression and other machine learning algorithm models were studied to assess the accuracy of BPM in different scenarios. Zhang et al. (30) evaluated the accuracy of linear regression, support vector regression, support vector machine (SVM) random forest regression and adaptive boosting machine learning models for BP prediction by extracting PTT, pulse wave characteristics and heart rate variability from two public datasets (MIMIC I and VitalDB) involving different populations and large sample sizes. Aguirre et al. (86) estimated the average ABP trend on samples from the MIMIC-III dataset *via* the deep learning model based on the seq2seq structure and by adding attention mechanisms using the pulse wave waveform. Without allowing data from the same subjects to be trained and tested, mean absolute error (MAE) of DBP met the requirements of AAMI with MAE of SBP being larger. These studies used public databases in model training to evaluate the accuracy of BP prediction in cases of multiple populations and samples. Machine learning methods include SVM, regression tree (RT), adaptive boosting (AdaBoost), and artificial neural network (ANN). Deep learning methods include convolution neural network (CNN), recursive neural network (RNN), and long-short-term memory (LSTM). Through overall processing of feature extraction and model establishment, it provides an opportunity for the use of single-channel PPG signals of the finger to achieve accurate BP prediction, and during this process calibration technology or strategy is needed in the current researches to measure the absolute BP.

## Discussion and conclusion

### Fusion method of the oscilloscope and photoplethysmography wave

In addition to main BPM technologies summarized in Table 1, some new detection methods integrate or optimize several traditional methods. The goal is to achieve miniaturization designs under the premise of ensuring accuracy. Studies have evaluated the medical-level BPM technology that combines the oscillographic method with the photoelectric volume pulse wave PPG. The basic principle is to combine the time, amplitude and change rate of the photoelectric volume wave at the distal end (such as the finger end) during the filling and discharging process to assist in determining accurate positions of SBP and DBP in the pressure envelope of the oscillographic method. Shalom et al. (92) used finger light volume recording signals or electronic recording of Korotkoff sounds, and detected the first PPG or Korotkoff pulse during cuff deflation to reduce the error rate in the first pulse detection while improving the accuracy of SBP measurements. Chandrasekhar et al. (93, 94) extended the technical principle of providing pressure signals through airbag devices in oscillometry. By integrating pressure sensors on smartphones, a finger-driven cuff less extended oscillometry was developed. The BPM technology, and its accuracy were tested by small-scale laboratory verification.

### Development of material science in the blood pressure measurement technology

**FIGURE 5:**
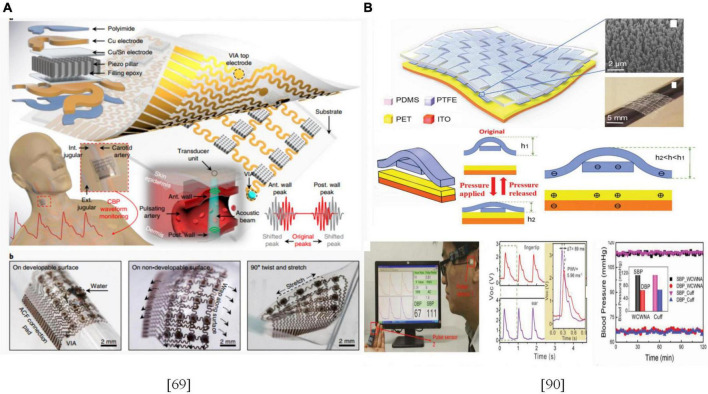
**(A)** Design and working principle of the stretchable ultrasonic device; **(B)** Schematic illustration of the flexible weaving self-powered pressure sensor and SBP/DBP measurements.

Advances in material science have provided new opportunities for miniaturization of BPM technologies. Liu et al. (88) developed a beat-to-beat BPM method that was based on arterial PTT and verified its feasibility by correlating the wavelength-dependent light penetration depth in the skin with skin blood vessels and using PTT on the skin arteriole to track peripheral resistance. The sensors used to detect physiological parameters, such as ECG, pulse and cardiac shock had flexible and bendable characteristics, which were associated with better adaptability and detection sensitivity (89–91). Wang et al. (70) developed a stretchable skin electronic device. Applications of the ultrasound technology to detect pressure waveforms of deep blood vessels can accurately monitor cardiovascular events from multiple body parts, which is expected to be used in multiple scenarios as displayed in Figure 5.

In summary, various BPM technologies in forms of wrist watches or finger rings are accurate in BPM on the wrist or fingers. These technologies have significant effects from the perspective of scientific research and engineering implementation, and can be used for BPM in various scenes under natural conditions in daily life. Although there have been significant advances in development and applications of remote non-invasive BPM technologies, some of the limitations in the existing finger-end BPM technologies include: i. Hand temperatures significantly affect signal quality, leading to measurement errors; ii. For users with peripheral arterial diseases, the reliability of measurement result is low; iii. Measurement errors can not negligible as a result of exercise interference and heart rate abnormalities. The challenges associated BPM technologies in the wrist are: i. The volume pulse wave perfusion ratio and signal-to-noise ratio of the wrist are low, and the stability is poor; ii. Pressure pulse wave detection of wrist radial artery and ulnar artery is susceptible to interference, which requires a high accuracy of detection position; iii. Affected by physiological structures, closure conditions of wrist arteries are more challenging than those of brachial arteries. Studies on new detection technologies to overcome these limitations and challenges will promote the development of daily multi-scene and multi-frequency BPM technologies, and provide the basis for realization of long-term personal BP management strategies.

The BP calculation method that is based on PTT or pulse wave morphological characteristics is mainly founded on the fluid mechanics or statistical models, in which determination of model parameters is mostly dependent on empirical values. Therefore, measurement accuracy should be clinically improved. Due to the limited number of model coefficients, establishment of coefficients that are suitable for the population through population data and adjustment of the coefficient to be suitable for a single individual through several calibrations is a major challenge. Although machine learning/deep learning model-based methods can reduce the detection signals of a channel on the basis of PTT, its calculation is large. To achieve better test accuracy in current laboratory research, it is necessary to set a strict standard for data selection. The signal quality of the original data should be high, however, in actual scenes, meeting these requirements are challenging.

Therefore, we postulate that: i. Medical-level oscillographic detection technologies should be designed based on characteristics of the detection site in the miniaturization process. A new type of airbag/cuff structure is designed or a flexible pressure sensor is used as a reference pressure source. Another signal channel that characterizes blood vessel states, such as volume pulse wave, should be introduced as a control quantity during pressure changes of the oscillographic method to achieve the goal of detecting oscillographic BP at the wrist or fingertip. The advantage of this technical method is that the low-load design of the device can integrate BPM into various daily life scenes, which is helpful to achieve high-precision BP monitoring with a long life cycle. ii. The PTT method is one of the hot spots in current research. It has obvious advantages in BPM in various scenes of daily life. However, its accuracy and physiological basis should be fully evaluated. By using the existing public datasets with BP calibration per blog, construction of reasonable and interpretable BP models will improve measurement accuracy and realize scientific prediction as well as tracking of changes in BP trends. In this process, construction of clinically calibrated datasets that can reflect BP changes in various scenarios in daily life will be of great significance in development of this method.

## Author contributions

YH and HW supervised the project. ZL and YH wrote the first draft of the manuscript. All authors contributed to writing and finalizing the manuscript and approved the submitted version.

## Abbreviations

BP: blood pressure
ABPM: ambulatory blood pressure monitoring
HBPM: home blood pressure monitoring
BPM: blood pressure measurement
PID: proportional-integral-derivative
FPS: flexible piezoresistive sensor
PTT: pulse transit time
PWV: pulse wave velocity
PAT: pulse arrival time
SBP: systolic blood pressure
DBP: diastolic blood pressure
NCBP: non-contact sphygmomanometer
MAE: mean absolute error
SD: standard deviation error
RMSE: root mean square error
SVM: support vector machine
RT: regression tree
AdaBoost: adaptive boosting
ANN: artificial neural network
CNN: convolution neural network
RNN: recursive neural network
LSTM: long-short-term memory
kNN: k-nearest neighbor
WkNN: weighted k-nearest neighbor
PPG: photoplethysmography
ECG: electrocardiograph.
